# Acute respiratory failure due to pulmonary exacerbation in children with cystic fibrosis admitted in a pediatric intensive care unit: outcomes and factors associated with mortality

**DOI:** 10.1186/s12931-024-02778-2

**Published:** 2024-04-29

**Authors:** David Drummond, Charlotte Roy, Matthieu Cornet, Julie Bucher, Véronique Boussaud, Françoise Le Pimpec-Barthes, Margaux Pontailler, Olivier Raisky, Vanessa Lopez, Claudio Barbanti, Romain Guillemain, Sylvain Renolleau, Marion Grimaud, Mehdi Oualha, Laure de Saint Blanquat, Isabelle Sermet-Gaudelus

**Affiliations:** 1 Unité de Transplantation Pulmonaire Pédiatrique, Hôpital Necker Enfants Malades, Assistance Publique Hôpitaux de Paris, Paris, France; 2 Centre Maladies rares Mucoviscidose et maladies apparentées, Hôpital Necker Enfants Malades, Assistance Publique Hôpitaux de Paris, Paris, France; 3 Université de Paris, Paris, France; 4 Institut Necker Enfants Malades, INSERM U1151, CNRS, Université de Paris, Paris, France; 5 CBIO-Centre de BioInformatique. Ecole des Mines, Paris, France; 6 Service de réanimation médico-chirurgicale pédiatrique, Hôpital Necker Enfants Malades, Assistance Publique Hôpitaux de Paris, Paris, France; 7 Hôpital Européen George Pompidou, Assistance Publique Hôpitaux de Paris, Paris, France; 8 Service de chirurgie thoracique et cardio-vasculaire pédiatrique, Hôpital Necker Enfants Malades, Assistance Publique Hôpitaux de Paris, Paris, France; 9 Service d’anesthésie et réanimation pédiatrique, Hôpital Necker Enfants Malades, Assistance Publique Hôpitaux de Paris, Paris, France

**Keywords:** Lung transplant, Mechanical invasive ventilation, Pediatrics, Chronic respiratory failure

## Abstract

**Background:**

Children with advanced pulmonary disease due to cystic fibrosis (CF) are at risk of acute respiratory failure due to pulmonary exacerbations leading to their admission to pediatric intensive care units (PICU). The objectives of this study were to determine short and medium-term outcomes of children with CF admitted to PICU for acute respiratory failure due to pulmonary exacerbation and to identify prognosis factors.

**Methods:**

This retrospective monocentric study included patients less than 18 years old admitted to the PICU of a French university hospital between 2000 and 2020. Cox proportional hazard regression methods were used to determine prognosis factors of mortality or lung transplant.

**Results:**

Prior to PICU admission, the 29 patients included (median age 13.5 years) had a severe lung disease (median Forced Expiratory Volume in 1 s percentage predicted at 29%). Mortality rates were respectively 17%, 31%, 34%, 41% at discharge and at 3, 12 and 36 months post-discharge. Survival rates free of lung transplant were 34%, 32%, 24% and 17% respectively. Risk factors associated with mortality or lung transplant using the univariate analysis were female sex and higher pCO2 and chloride levels at PICU admission, and following pre admission characteristics: home respiratory and nutritional support, registration on lung transplant list and *Stenotrophomonas Maltophilia* bronchial colonization.

**Conclusion:**

Children with CF admitted to PICU for acute respiratory failure secondary to pulmonary exacerbations are at high risk of death, both in the short and medium terms. Lung transplant is their main chance of survival and should be considered early.

## Introduction

Cystic fibrosis (CF) is a multiorgan disease that affects primarily the lungs, causing diffuse bronchiectasis whose evolution leads progressively to respiratory failure [1, 2]. Although Highly Effective Therapeutic Modulators have considerably improved the prognosis of people with CF, patients carrying non-eligible genotypes are still at risk of developing severe lung disease. Outcomes of adult patients with CF admitted to Intensive Care Unit (ICU) for acute respiratory failure have improved consistently over the past 30 years. In the 1980s, ICU admission was controversial since it was associated with a high mortality rate (69% in ICU; 81% at one year after discharge) especially when the patients required endotracheal intubation [3]. With the increased implementation of non-invasive ventilation (NIV), ICU mortality rates decreased to 32–55% in the 1990s [4] and 14% in the 2000s [4–7]. Regarding the most severe patients requiring invasive mechanical ventilation (IMV), survival rates also largely improved with more than half of the patients being discharged in the last study [8–11].

By contrast, the different outcomes for children with CF admitted to pediatric intensive care unit (PICU) for acute respiratory failure have been poorly studied. Only 5 studies focused specifically on this population [12–17]. The most recent study reported an overall mortality rate of 6.6% for children hospitalized between 2009 and 2018 in 135 PICUs [17]. This improved outcome also reflects the progress in survival of the CF population overall as well as proactive supportive care in these severe patients. Indeed, advances in critical care interventions, including IMV strategies and extracorporeal membrane oxygenation (ECMO) as bridge to transplant likely contributed also to the improvement in the overall mortality of these critically ill CF children [18].

Paradoxically, short and longer-term outcomes at discharge from PICU have not been reported. We hypothesized that admission in PICU for respiratory failure is a risk factor of death or terminal respiratory decompensation requiring lung transplant in CF children. To answer this question, we performed a retrospective study to analyze the different outcomes for children and adolescents with CF admitted to PICU for acute respiratory failure and identify mortality or lung transplant associated factors during a post PICU 36 month-follow up.

## Methods

### Study population

This retrospective single center study was conducted in the PICU of the University Hospital Necker-Enfants Malades (Paris, France). This hospital includes a center specialized in CF and is a referral hospital for pediatric lung transplant. All CF patients admitted to PICU for respiratory failure due to respiratory exacerbation between 1/1/2000 and 1/1/2020 were identified using electronic health records of the department. Patients were followed-up for 36 months after their admission in ICU, to a maximal data point of 31/12/2021. Children admitted for acute respiratory failure related to hemoptysis, pneumothorax and other rapidly reversible causes were excluded from the analysis. This study has been declared to the APHP clinical research depository in accordance with Human Ethics and Consent to Participate. Patients have been informed of the potential use of their clinical data for research.

### Data collection

Data related to the ICU admission were collected from the electronic health records of the department. Data related to CF history and follow-up after ICU admission were collected from the local database. This included:

(i) CF history: registration on lung transplant waiting list, hemoptysis, pneumothorax, home ventilatory support (oxygen therapy and/or non-invasive ventilation (NIV)), nutritional support (enteral and/or parenteral nutrition), airway chronic bacterial colonization, number of intravenous and oral antibiotic courses, number of hospitalizations, extra-pulmonary involvement (CF related diabetes and impaired glucose tolerance [19], CF related hepatopathy [20], Broncho Pulmonary Aspergillosis [21], pulmonary hypertension (defined by average pressure in the pulmonary artery at more than 25mmHg) [22].
(ii) The severity of respiratory insufficiency was assessed by (i) the best baseline lung function performed during a stable outpatient visit within the year before ICU admission using Forced Expiratory Volume in 1 s (FEV_1_), and Forced Vital Capacity (FVC)values. Both FEV_1_ and FVC were expressed as percentage of predicted value for age, sex and height according to Knudson normal values (FEV_1_pp, FVCpp), (ii) the slope in lung function decline in the last 12 months, calculated from available lung function tests recorded within the 12 months before ICU admission.
(iii) Characteristics upon admission in ICU: age, sex, body mass index (BMI), biological blood parameters (pH, pCO_2_, bicarbonate, sodium, chloride, C-Reactive Protein (CRP)) and respiratory support (non-invasive or invasive ventilation during hospitalization). Acute respiratory failure was defined as (i) hypoxemia (SpO2 < 92% in room air), (ii) a PCO2 > 65 mm Hg or 20 mm Hg above basal level and (iii) respiratory support (or increased need) (non invasive or invasive ventilation).
(iv) Patient outcome after PICU admission, and 3, 12 and 36 months after admission: death, lung transplant in the frame of the High Emergency Lung Transplantation during ICU, lung transplant after discharge and survival.

### Study endpoint

The primary study endpoint was survival free of lung transplantation at PICU discharge. The second study endpoint was survival free of lung transplantation during the 36 months of the follow-up.

### Statistical analysis

Analyses were conducted with the R software (R-4.3.2 for Windows) [23]. Results are presented as median values and interquartile ranges. Comparisons of the characteristics of the patients according to their outcome were made using nonparametric statistical methods (Wilcoxon’s and Fisher’s exact test tests) because of the non-normal distribution of several variables and the small numbers of patients in the groups of interest. Results of univariate analysis are expressed using the hazard ratio (HR) and 95% confidence interval (CI).

Cox proportional hazard regression methods were used to determine the association between patient characteristics and outcome during the 36 months follow-up.

## Results

### Population

During the period of the study, all causes included, 50 patients with CF were admitted in PICU. Among them 29 of the 50 patients (58%) were admitted for acute respiratory failure and included in the study. The characteristics of the patients are presented in Table 1. Age ranged from 6 to 17 years. Children had a severe CF respiratory disease based on FEV_1_pp. Children had a chronic colonization with *P.Aeruginosa* (86%) *Aspergillus species* (51%) and Methicillin-resistant *S.Aureus* (41%). 21 of the 29 patients (72%) had home NIV. BMI z-core ranged between − 3.4 and 1.3 and 72% of the children had enteral nutrition support. Only 14 (48%) of those children were on lung transplant list at admission in the ICU. No patients were treated with CFTR modulators because either they were enrolled before availability of modulators or their genotype was not eligible. The median number of antibiotic IV course was 6.5 (0–12) during the year previous to the admission.

**Table 1:** Characteristics of patients at admission in PICU, presented as median (interquartiles)

**General characteristics**	
Age, year	13.5 (6.2–17.5)
Sex, male/female	10/19
**Genotype** ^**a**^	
F508del/ F508del	13 (45%)
F508del /MF	9 (31%)
MF/ MF	6 (21%)
**Nutritional status**	
Body mass index (Z-Score) 1 year before PICU	–0.94 (-3.4 to + 1.3)
Enteral nutrition	21 (72%)
Parenteral nutrition	4 (14%)
Chronic central venous catheter	25 (8%)
**Pulmonary involvement**	
History of hemoptysis	12 (41%)
History of pneumothorax	3 (10%)
**Lung function 1 year before PICU**	
FEV_1_pp	29 (13–56)
FVCpp	41 (15–67)
**Lung function decline in the previous year**	
FEV_1_pp	–2 (-25 to + 8)
FVCpp	–5 (-44 to + 9)
Supplemental oxygen	22 (76%)
Non-invasive ventilation	21 (72%)
**Patients awaiting lung transplant**	14 (48%)
**Chronic airway colonization**	
*P. Aeruginosa*	25 (86%)
*B. Cepacia complex*	4 (14%)
*A. Xylosoxydans*	6 (21%)
Methicillin-sensitive *S. Aureus*	8 (32%)
Methicillin-resistant *S. Aureus*	12 (41%)
*Aspergillus* species	15 (52%)
*S. Maltophilia*	4 (14%)
**Comorbidities**	
CF-related diabetes	9 (31%)
CF liver disease	13 (45%)
Pulmonary hypertension	5 (17%)
BronchoPulmonary Aspergillosis	6(21%)

### Outcome at PICU discharge

Upon admission in PICU, all patients required oxygen therapy, 27 (92%) had a NIV and 3 (10.3%) had invasive ventilation (Table 2).

**Table 2:** Characteristics of patients according to their status at discharge of PICU, presented as median (interquartiles)

	Survival at discharge (*n* = 10)		Death or lung transplant (*n* = 19)		p		
**Before PICU hospitalization**							
**General characteristics**							
Age, year	12.2 (9-15.3)		13.1 (6.2–17.5)		0.21		
Sex, (male/female)	8/2		2/17		< 0.01		
**Nutritional status**							
Body mass index (Z-Score)	−0.6 (-2.5 to + 1.3)		–1.1 (-3.4 to + 0.5)		0.37		
Enteral nutrition	5 (50%)		16 (84%)		0.13		
Parenteral nutrition	0 (0%)		4 (21%)		0.32		
**Pulmonary involvement**							
**Lung function (1 year before)**							
FEV_1_pp	36 (17–56)		27 (13–49)		0.09		
FVCpp	45 (15–67)		38 (18–55)		0.50		
**Lung function decline in the previous year**							
FEV_1_pp	–5.8 (-25 to + 2.0)		–4.2 (-18.0 to + 8.0)		0.95		
FVCpp	–9.7 (-44.0 to + 9.0)		–6.0 (-29.0 to + 8.0)		0.76		
**Ventilation support**							
Supplemental oxygen	6 (60%)		16 (84%)		0.32		
Non-invasive ventilation	5 (50%)		16 (84%)		0.13		
**Chronic airway colonization**							
*P. Aeruginosa*	8 (80%)		17 (89%)		0.89		
*B. Cepacia complex*	1 (10%)		3 (16%)		1		
*A. Xylosoxydans*	1 (10%)		5 (26%)		0.58		
Methicillin-sensitive *S. Aureus*	4 (40%)		6 (32%)		0.97		
Methicillin-resistant *S. Aureus*	5 (50%)		6 (32%)		0.57		
Aspergillus species	6 (60%)		9 (47%)		0.80		
*S. Maltophilia*	0 (0%)		4 (21%)		0.32		
**Comorbidities**							
CF-related diabetes	4 (40%)		5 (26%)		0.74		
**At arrival in PICU**							
**Respiratory support**							
Non-invasive ventilation		10 (100%)		17 (89%)		0.77	
Invasive ventilation		0 (0%)		3 (11%)		0.49	
**Nutritional status**							
Body mass index (Z-Score)		–0.3 (-2.0 to + 1.7)		–0.9 (-2.4 to + 0.9)		0.21	
**Biological parameters**							
pH		7.39 (7.26–7.47)		7.34 (7.25–7.44)		0.04	
pCO_2_		57 (39–101)		77 (41–112)		< 0.01	
Bicarbonates		33 (22–61)		38 (29–51)		0.02	
Sodium		134 (121–142)		137 (130–144)		0.32	
Chlorides		93 (71–100)		90 (80–101)		0.19	
CRP		81 (6-215)		117 (7-328)		0.49	
**Respiratory support in PICU**							
Non-invasive ventilation only		9 (90%)		12 (63%)		0.27	
Mechanical invasive ventilation		1 (10%)		9 (47%)		0.11	

Of the 29 patients admitted in PICU, 5 (17%) died during their PICU stay from refractory respiratory failure and 10 (35%) were discharged free from lung transplantation. Fourteen of the 29 patients (48%) received lung transplant during the PICU stay and among those, 12 in the frame of the High Emergency Lung Transplantation (HELT) French program.

**Fig. 1:**
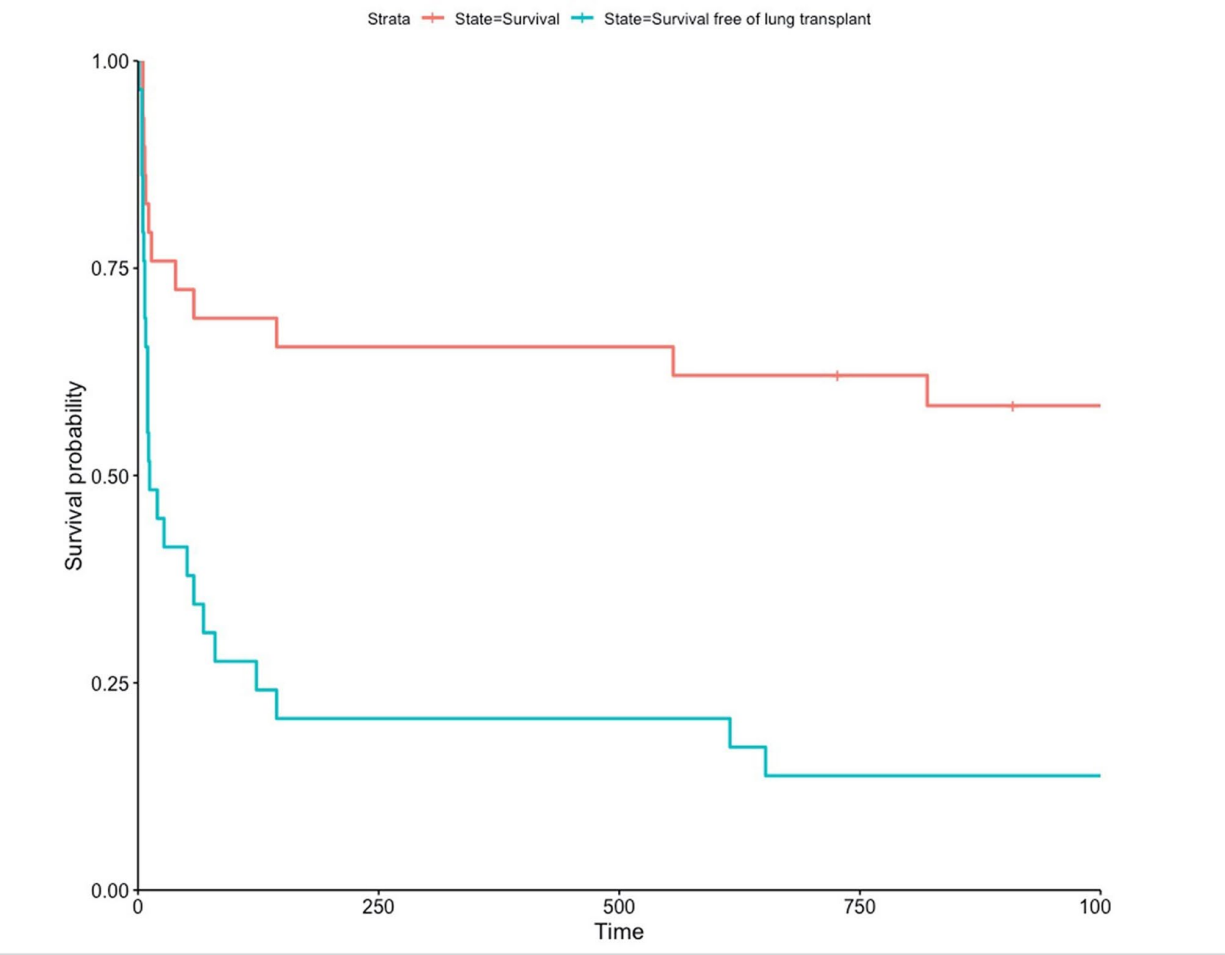
Survival free of lung transplantation and overall survival after PICU admission

Follow up after discharge ranged between 3 months to 12 years and transplant-free survival is illustrated in Fig. 1.

Among the 10 non-transplanted survivors at discharge from the PICU, 3 patients (30%) required lung transplant during follow up and 3 patients (30%) were alive without lung transplant after a 36 months follow up. Indication for transplant was terminal respiratory failure and occurred at 4, 17 and 36 month after discharge.

Among the 14 patients transplanted during the PICU stay, 3 died within 3 months post-transplant (respectively at 8, 14 and 39 days post-transplant, from multiorgan failure), and 2 after one year and a half post-transplant (18 months and 26 months respectively from chronic lung allograft dysfunction). One patient over 29 (3%) required ECMO as a bridge to transplant.

**Fig. 2:**
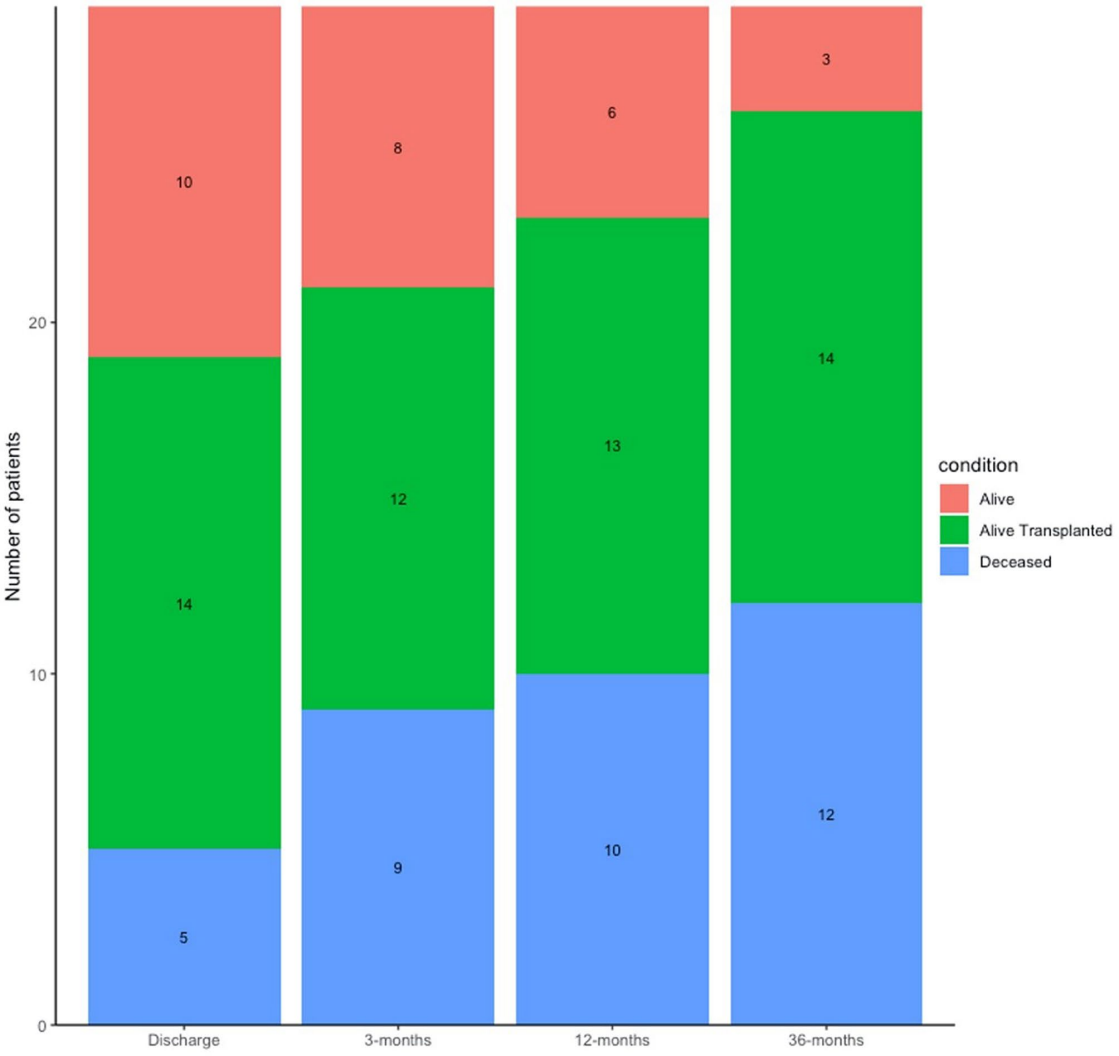
Outcomes at discharge, 3 months, 12 months and 36-months after PICU admission

Overall, regarding the 29 patients, the mortality rate was 31% at 3 months, 34% at 12 months and 42% at 36 months. Among the 17 patients still alive at 36 months (58%), 12 had had lung transplantation (70%). From the 17 transplanted patients, 5 were dead within the 36 months after transplant (Fig. 2).

### Factors associated with death or lung transplant

Characteristics of patients according to their status at discharge of PICU are shown in Table 2. pCO_2_ and bicarbonate levels upon arrival were significantly higher among the patients dying during ICU. The probability of death was significantly increased in females. The results of the univariate proportional hazard assessment of risk factors for death and lung transplantation are presented in Table 3.

**Table 3:** Factors associated with mortality or lung transplant – univariate analysis

Factor	Hazard Ratio	[95% CI]	p-value
**General characteristics**			
Age, year	1.1	[0.9–1.3]	0.29
Sex, (female)	6.6	[2.1–21]	**< 0.01**
**Nutritional status**			
Body mass index (Z-Score)	0.76	[0.6-1.0]	0.09
Enteral nutrition	3.2	[1.1–9.1]	**0.03**
Parenteral nutrition	6.1	[1.8–20]	**< 0.01**
Chronic central venous catheter	3.6	[0.8–15]	0.09
**Pulmonary involvement**			
History of hemoptysis	1.3	[0.6–2.8]	0.56
History of pneumothorax	1.5	[0.4–5.1]	0.52
Lung function (1 year before)			
FEV_1_pp	0.97	[0.9-1.0]	0.15
FVCpp	0.98	[1.0–1.0]	0.22
**Lung function decline in the previous year**			
FEV_1_pp	1.0	[1.0-1.1]	0.67
FVCpp	1.0	[1.0–1.0]	0.93
**Ventilation support**			
Supplemental oxygen	3.2	[1.1–9.5]	**0.04**
Non-invasive ventilation	3.6	[1.3–10]	**0.02**
**Patients awaiting lung transplant**	4.8	[1.8–13]	**< 0.01**
**Chronic airway colonization**			
*P. Aeruginosa*	2.7	[0.6–12]	0.17
*B. Cepacia complex*	2	[0.7–6.1]	0.20
*A. Xylosoxydans*	1.1	[0.4–2.9]	0.88
Methicillin-sensitive *S. Aureus*	0.84	[0.4–1.9]	0.67
Methicillin-resistant *S. Aureus*	0.6	[0.3–1.4]	0.24
Aspergillus species	0.79	[0.4–1.7]	0.55
*S. Maltophilia*	4.1	[1.2–14]	**0.02**
**Comorbidities**			
CF-related diabetes	1.2	[0.5–2.7]	0.73
CF liver disease	1.2	[0.5–2.6]	0.69
Pulmonary hypertension	0.82	[0.3–2.4]	0.71
**Nutritional status on arrival at PICU**			
Body mass index (Z-Score)	0.81	[0.6–1.1]	0.24
**Biological parameters on arrival at PICU**			
pH	0.002	[0-1.3]	0.06
pCO2	1.0	[1.0-1.1]	**< 0.01**
Bicarbonates	1.0	[1.0-1.1]	0.06
Sodium	1.1	[1.0-1.2]	0.1
Chlorides	0.95	[0.9-1]	**0.04**
CRP	1	[1.0–1.0]	0.06
**Respiratory support in PICU**			
Non-invasive ventilation only	0.03	[0-0.2]	**< 0.01**
Mechanical invasive ventilation	2.4	[0.7–8.1]	0.17

Factors significantly associated with increased mortality or lung transplant were related to CF severity (home Oxygen, home NIV, nutritional support, registration on transplant list) and biological markers upon admission in ICU (higher pCO_2_, lower chloride level). In contrast NIV was associated with significant decreased risk of mortality and lung transplant (0.03 (0-0.2)). Colonization with *S.Maltophilia* before transplant increased the risk by 4.1 (1.2–14). Importantly, the female sex increased the risk of a bad outcome by 6.6 (2.1–21). Lower FEV_1_pp or BMI, P*seudomonas Aeruginosa* and *Burkholderia Cepacia* colonization and mechanical ventilation during ICU increased the risk of death or lung transplant at short term but all those factors did not reach significance.

Neither age, history of hemoptysis or pneumothorax, lung bacterial or fungal infection, kinetics of decline in respiratory function or nutritional status were significantly associated with mortality or lung transplant. Additionally, comorbidities including diabetes, pulmonary hypertension, liver disease were not associated with increased mortality.

## Discussion

To our knowledge, this is the first study to investigate long term outcomes of children with CF admitted to PICU for respiratory failure. Two main conclusions can be drawn from this study: [1] Admission in PICU for acute respiratory failure is associated with poor outcomes, with only 35% of children being discharged alive without lung transplantation; [2] Referral to a lung transplant center must be considered for those surviving to the PICU stay as 36 months after discharge only 30% were still alive without lung transplantation and 60% deceased.

Mortality among our population of CF children with respiratory failure hospitalized in PICU was 17%. This is significantly higher than the number of 6.6% reported by Smith et al. [17]. However this latter study did not focus on patients hospitalized for respiratory failure. As our main question was to refine indications for short term lung transplant indication in CF children hospitalized in PICU, we focused on patients with respiratory failure due to pulmonary exacerbation and excluded rapidly reversible causes, as these conditions are associated with a better prognosis [8, 11].

In our study, main risk factors of mortality or lung transplantation during PICU were related to respiratory failure as shown by the level of pCO_2_ and Bicarbonate significantly more elevated in the group with pejorative evolution. Lower FEV_1_ and BMI, *P.Aeruginosa* and *B.Cepacia* colonization, mechanical ventilation during ICU did not reach significance probably because patients were already very severe and, most importantly, the study lacked power.

Death or lung transplant during the 36 months following PICU admission were mainly linked to chronic respiratory failure, as assessed by an increased risk in patients with home supplemental oxygen or non-invasive ventilation, and in those already on transplant list. This is also supported by the observation that patients with a poor evolution had increased levels of bicarbonate and decreased level of chloride at hospitalization which reflects chronic respiratory acidosis with increased kidney bicarbonate reabsorption and decreased chloride reabsorption [24]. However a renal CFTR dependent participation in the regulation of bicarbonate serum level cannot be excluded [25]. *S.Maltophilia* lung infection was significantly more prevalent in children with a bad evolution and may be an additional pejorative associated factor. *S Maltophilia* has been reported in association with low FEV_1_ in children, however, epidemiological data are still needed to confirm the pathogenicity of this microorganism [26].

Indeed, it is still unclear whether *Stenotrophomoas Maltophilia* per se has a pathogenic role or is a biomarker of the disease. Terlizzi et al. reported in a recent review that this bacterium is found in patients with respiratory decline while several previous studies did not evidence any impact for *S. Maltophilia* in the respiratory status evolution [27]. Interestingly enough, recent studies in patients with milder disease provide evidence that *S.Maltophilia* intermittent or chronic colonization might be a factor of respiratory degradation [26, 28]. More specifically in *Pseudomonas aeruginosa* negative patients, the presence of *S.Maltophilia* and most importantly its acquisition are significantly associated with FEV_1_ decline in a multivariate analysis [28, 29]. Considering our results, in severe patients, it is reasonable to attempt to eradicate this microorganism, as proposed by Terlizzi et al. [27]. Finally, patients with home nutritional support were also at increased risk of death or lung transplant, probably reflecting the increased energy expenditure frequent in patients with CF and chronic respiratory failure [30]. Each of these factors could be considered markers of more severe underlying diseases, highlighting the importance of longitudinal disease control and proactive early care. Similarly, Smith et al. also reported increased odds of mortality in patients with features of severe respiratory failure such as hemoptysis, pneumothorax, bacterial/fungal infections, malnutrition, and need for noninvasive or invasive respiratory support [17]. Our results are also consistent with those reported by Zolin et al using European Cystic Fibrosis Society Patient Registry database of 24,625 patients [31]. Indeed, although, the percentage of deceased children is higher in countries with low/middle income than those with higher income as France, FEV1pp below 40% and BMI zscore below − 2 are significant risk factors of childhood mortality in this case control retrospective registry study in either income group. Those parameters were not significantly associated with death in our study, both because of the lack of power in this small sample but also because the population in our study was restricted to patients hospitalized in ICU because of respiratory failure. We confirm the role of the female sex, and interestingly point, *S.Maltophilia* as an additional predictive biomarker of severity.

Upon arrival in PICU, higher levels of pCO_2_ and MIV were also associated with poor outcomes, as already described in adults, while children undergoing NIV were at significantly lower risk of pejorative outcome [19, 32]. This suggests that NIV should be tried in a first line approach for those patients instead of mechanical ventilation. Malnutrition and any other comorbidities were not associated with increased mortality in our cohort. This is unexpected as prior studies did show a direct correlation between BMI and outcomes such as mortality and pulmonary function in CF patients [30]. However, as a matter of fact, most of our patients had nutrition support and their BMI was within the normal range. Finally, female sex was also identified as a risk factor. This is in line with previous publications that reported significantly higher mortality in female subjects [33, 34], although it is not a consistent finding [35].

Children with CF enrolled in this study often received a lung transplant during their PICU hospitalization. Those admitted after July 2007 benefited from the implementation of the HELT system that prioritizes graft allocation to patients with short-term severe prognoses. This new organ allocation system was not associated with higher post-transplant mortality in adults, with a survival rate at 12 months around 80% [36, 37]. Twelve children of our cohort were transplanted within the frame of HELT and their survival at 12 months was comparable to that of adults.

Most importantly, our results show very low rates of survival without lung transplantation (17%) during the 36 months following PICU admission for children with chronic respiratory insufficiency. Lung transplantation should therefore be considered early after PICU admission and children referred to lung transplant centers immediately after their discharge. A study conducted in France found that 80% of CF patients who died between 2007 and 2010 were not on any lung transplantation waiting list, and that this lack of listing was primarily related to late or lack of transplantation referral, rather than contraindication to transplantation [38]. Our study unveils a similar observation as only half of the children in our cohort were on the waiting list for lung transplantation. The CF Foundation recommends lung transplant referral in children and adolescents to be anticipated no later than when FEV_1_pp is < 40% = or FEV_1_ is < 50% and rapidly declining or associated with markers of shortened survival including hypoxemia, hypercapnia, pulmonary hypertension, malnutrition, multiple exacerbations, massive hemoptysis and pneumothorax [39]. We state that children on enteral nutritional support, chronic oxygen or NIV at home should be referred for lung transplants, all the more they are female and colonized with *S.Maltophilia*.

This study has several limitations. Its retrospective design precludes conclusions regarding causality between the factors identified and the risk of mortality or lung transplantation. The small number of patients limits the statistical power of the analysis conducted. The external validity of this monocentric study needs to be confirmed in other centers, especially since our center is a referral hospital for CF patients registered on the lung transplant waiting list and therefore admits the most severe patients from several regions in France. Finally, within such an extended timeframe, changes in CF care such as implementation of extracorporeal membrane oxygenation [40], and the advent of CF modulators [41], may jeopardize the data.

## Conclusion

In conclusion, this study found that children and adolescents with CF admitted to PICU for respiratory failure secondary to pulmonary exacerbations were at high risk of death. Lung transplantation is their main chance to survive for those discharged alive without lung transplantation. Children with CF who need critical care because of decompensation of a endstage respiratory failure need to be transplanted in short-term period.

## Data Availability

Data is provided within the manuscript.

## Abbreviations

CF: cystic fibrosis
FEV1: Forced expiratory volume in one second
ICU: intensive care unit
MIV: mechanical invasive ventilation
NIV: non-invasive ventilation
PICU: pediatric intensive care unit
MF: Minimal Function associated with this CFTR genotype
